# Assessing AI-Generated Autism Information for Healthcare Use: A Cross-Linguistic and Cross-Geographic Evaluation of ChatGPT, Gemini, and Copilot

**DOI:** 10.3390/healthcare13212758

**Published:** 2025-10-30

**Authors:** Salih Rakap, Emrah Gulboy, Uygar Bayrakdar, Goksel Cure, Busra Besdere, Burak Aydin

**Affiliations:** 1 Department of Specialized Education Services, School of Education, University of North Carolina Greensboro, Greensboro, NC 27412, USA; 2 Developmental Education Application and Research Center (OGEM), Ondokuz Mayis University, Samsun 55270, Türkiye; emrah.gulboy@omu.edu.tr (E.G.); uygar.bayrakdar@omu.edu.tr (U.B.); 3 Department of Special Education, School of Education, Giresun University, Giresun 28200, Türkiye; gokselcure@hotmail.com; 4 Manchester Institute of Education, The University of Manchester, Manchester M13 9WJ, UK; busra.besdere@manchester.ac.uk; 5 Department of Educational Sciences, School of Education, Ege University, İzmir 35100, Türkiye; 6 Department of Educational Sciences, School of Education, Leuphana University, 21335 Lüneburg, Germany

**Keywords:** autism, Artificial Intelligence (AI), healthcare communication, Large Language Models (LLMs), ChatGPT, Copilot, Gemini

## Abstract

**Background/Objectives:** Autism is one of the most prevalent neurodevelopmental conditions globally, and healthcare professionals including pediatricians, developmental specialists, and speech–language pathologists, play a central role in guiding families through diagnosis, treatment, and support. As caregivers increasingly turn to digital platforms for autism-related information, artificial intelligence (AI) tools such as ChatGPT, Gemini, and Microsoft Copilot are emerging as popular sources of guidance. However, little is known about the quality, readability, and reliability of information these tools provide. This study conducted a detailed comparative analysis of three widely used AI models within defined linguistic and geographic contexts to examine the quality of autism-related information they generate. **Methods:** Responses to 44 caregiver-focused questions spanning two key domains—foundational knowledge and practical supports—were evaluated across three countries (USA, England, and Türkiye) and two languages (English and Turkish). Responses were coded for accuracy, readability, actionability, language framing, and reference quality. **Results:** Results showed that ChatGPT generated the most accurate content but lacked reference transparency; Gemini produced the most actionable and well-referenced responses, particularly in Turkish; and Copilot used more accessible language but demonstrated lower overall accuracy. Across tools, responses often used medicalized language and exceeded recommended readability levels for health communication. **Conclusions:** These findings have critical implications for healthcare providers, who are increasingly tasked with helping families evaluate and navigate AI-generated information. This study offers practical recommendations for how providers can leverage the strengths and mitigate the limitations of AI tools when supporting families in autism care, especially across linguistic and cultural contexts.

## 1. Introduction

Autism is one of the most frequently diagnosed neurodevelopmental conditions, affecting approximately one in every 31 children in the United Staes [1] and 1.7% of the population in the United Kingdom [2]. With rising prevalence rates, there has been an explosion of information about autism available online. Parents and caregivers increasingly rely on the internet as their primary source of information when navigating autism-related questions [3,4]. While this accessibility can empower families, the overwhelming volume of content often blends credible, evidence-based information with misinformation and myths. This conflation makes it difficult for individuals to discern accurate knowledge from outdated or false narratives [5,6]. Historically, autism-related misinformation has been pervasive, with debunked myths, such as the association between vaccines and autism or the “refrigerator mother” hypothesis, continuing to influence public understanding despite extensive scientific refutation [7]. Compounding this challenge is the tendency for reliable, evidence-based autism information to be presented in dense academic language, which may be inaccessible to the average reader. Thus, the need for platforms that provide clear, accurate, and easily understandable autism-related content is very critical [8].

From a theoretical standpoint, this challenge can be framed through the lens of eHealth Literacy [9], which refers to an individual’s ability to seek, understand, and evaluate health information from electronic sources and apply this knowledge to make informed decisions. Caregivers with limited eHealth literacy may struggle to interpret or verify online information, making them more vulnerable to misinformation or overly technical content [10]. In addition, Cognitive Load Theory [11,12] suggests that when information is presented in overly complex or lengthy formats, it can exceed the reader’s working memory capacity, thereby reducing comprehension and recall. Thus, readability is not only a stylistic concern but a cognitive and accessibility issue that directly influences health decision-making and digital trust [13,14].

Healthcare providers including pediatricians, psychiatrists, and other specialists play a central role in the early identification, diagnosis, and ongoing follow-up of children with autism [15]. In addition to delivering clinical care, they are often the first point of contact for parents seeking guidance about autism and are responsible for supporting families in accessing accurate information [16,17]. However, given time constraints in clinical practice and the complexity of autism-related resources, healthcare professionals cannot always provide detailed, ongoing education. In this context, accessible and reliable information from AI-driven platforms could complement providers’ efforts by reinforcing key messages, clarifying misconceptions, and offering families user-friendly explanations outside of clinical encounters [18].

The advent of artificial intelligence (AI)-driven conversational agents has introduced new opportunities for addressing information gaps, particularly in health-related domains. Tools such as ChatGPT, Gemini, and Microsoft Copilot, though originally designed for distinct purposes, are increasingly being explored as reliable sources of health information [19,20,21]. ChatGPT, developed by OpenAI, utilizes the Generative Pretrained Transformer (GPT) framework to deliver conversational and user-friendly responses [22]. Google’s Gemini combines advanced search capabilities with natural language processing to provide fact-based, context-aware answers [23]. Microsoft Copilot, part of Microsoft’s productivity ecosystem, generates contextually relevant, human-like content tailored to user queries [24]. Recent studies have begun to assess these tools’ capacity to deliver accurate, medically relevant content, particularly in specialized domains like pharmacology and cardiovascular imaging [25,26]. These studies have consistently found that ChatGPT outperforms Gemini and Copilot in accuracy and completeness, though differences often vary depending on the complexity of the question or domain.

Each tool brings unique strengths. ChatGPT is particularly effective at simplifying complex topics and making them more accessible for non-specialists, such as non-native speakers, individuals with reading difficulties [27]. Gemini benefits from being grounded in Google’s search infrastructure and data centers, which helps improve reliability and reduce errors [28]. Copilot, with integration of public web search and document sources, shows strength in producing streamlined, fact-based responses in technical or factual domains, though its performance may vary by language and content area [29]. However, these performance differences become more evident in educational and clinical settings, where narrative coherence and logical reasoning are essential. For example, Rossettini et al. [30] found that while ChatGPT and Copilot performed well on structured healthcare science exams, Gemini struggled to maintain consistent accuracy and coherence. These tools hold significant potential to improve access to accurate, user-friendly, and actionable information, especially in areas where reliable and accessible knowledge is critical, such as autism-related topics. Importantly, their use in healthcare contexts raises the question of how such tools might augment provider-patient communication by extending the reach of clinicians’ guidance into families’ everyday information-seeking practices. Given these patterns, comparative research focusing on model performance across languages and contexts is both timely and necessary to understand the practical relevance of AI-generated health communication.

However, these tools may rely on different underlying data sources, potentially leading to variations in the responses generated for the same questions. Furthermore, the information provided by these tools may differ depending on the user’s location and the language in which the query is posed, introducing additional variability in the results [31,32,33]. This variability underscores the importance of critically evaluating their outputs. Additionally, all three systems share common limitations, including the potential to disseminate inaccuracies, fabricate sources, or provide biased or incomplete information. Moreover, there is skepticism about whether these tools are equipped to navigate the nuances of autism-related topics, including dispelling persistent myths and offering evidence-based resources [3]. For healthcare providers, understanding both the strengths and weaknesses of these platforms is essential, since families may increasingly use AI-generated content to supplement or even replace professional advice [34].

The literature includes numerous studies examining the quality of information about autism presented on websites and other online platforms [4,35,36]. These studies have identified significant variability in the accuracy, completeness, and reliability of the information available, further highlighting the challenges faced by individuals seeking evidence-based resources. More recently, attention has turned to AI-driven tools such as ChatGPT as emerging sources of health-related information. Several studies have evaluated the quality of responses provided by ChatGPT on a range of health topics, including COVID-19 [21], mental health [20], and general medical knowledge [19]. However, research specifically focusing on autism-related content generated by AI tools remains sparse.

We found two studies in the literature focused on autism and the use of AI tools. McFayden et al. [3] evaluated ChatGPT’s responses to 13 autism-related questions, including basic information, myths, and resources. Their findings indicated that ChatGPT produced largely accurate, clear, and concise information, especially when debunking common misconceptions. However, limitations were noted in actionability, with few practical next steps provided, and reference accuracy, where many hyperlinks were outdated or non-functional. They concluded that ChatGPT is a viable tool for parents seeking autism-related information but requires improvements in actionable advice and citation reliability. Hassona and Alqaisi [37] focused on ChatGPT’s responses to 17 questions related to autism and oral health. They found that ChatGPT provided comprehensive, largely correct, and empathetic information, including practical tips for parents and recommendations for multidisciplinary care. However, the readability of the responses was beyond the recommended level for lay audiences, potentially limiting accessibility. Both studies highlight ChatGPT’s potential as a source of autism-related information while underscoring areas for improvement in usability, readability, and actionability. These findings warrant additional studies to explore the performance of AI tools in providing autism-related information across various contexts, languages, and user needs to fully understand their potential and limitations.

The present study builds on this emerging body of research through a detailed comparative analysis of three leading AI tools, ChatGPT, Gemini, and Copilot, within specific linguistic and geographic contexts. Specifically, we seek to investigate the accuracy, readability, actionability, language use, and reference quality of the information these tools generate. Our analysis focuses on responses to common autism-related questions parents may ask, spanning several key dimensions: understanding autism, diagnosis, risk factors and causes, prognosis and lifespan, treatment and interventions, skill development and behavior management, education and inclusion, and daily life and routines. Importantly, we will explore how these tools perform when accessed from different countries, Türkiye, England, and the United States (USA), and in different languages, namely Turkish and English. This comparative approach seeks to highlight the strengths and limitations of each tool while also evaluating how geographic location and language influence the quality, accuracy, and reliability of the information provided. By examining performance across two languages (English and Turkish) and three countries (the USA, England, and Türkiye), the study provides comparative depth on how language and location influence the quality, accuracy, and reliability of AI-generated autism information.

As a replication and extension of McFayden et al. [3], this study broadens the scope by including two additional AI tools, a larger pool of autism-related questions, systematic evaluation of readability, and analyses across multiple languages and geographic locations. In doing so, it provides a more thorough assessment of the usefulness of AI-generated information for healthcare providers across different regions and languages and its potential to serve as a complementary resource in clinical contexts to bridge gaps in autism education and parent support.

The study was guided by the following research questions:

To what extent does the accuracy of autism-related information generated by ChatGPT, Gemini, and Microsoft Copilot vary by geographic location (USA, England, Türkiye) and language (English vs. Turkish)?
How do ChatGPT, Gemini, and Copilot differ in the readability of their responses across languages and countries?
How likely are these tools to generate actionable guidance in response to caregiver-focused autism questions, and does this vary by language or domain?
What types of language framing (medicalized vs. neurodiversity-affirming) are used by each model, and how do these differ by location and language?
How do reference frequency, domain credibility, and hyperlink functionality differ across tools and contexts?

## 2. Method

### 2.1. Search Using the AI Platforms

This study employed a cross-linguistic and cross-geographic evaluation of three AI-driven conversational tools: ChatGPT 4, Gemini 2.0, and Microsoft Copilot. Data collection took place in May 2025. A total of 44 open-ended questions were organized across seven domains that reflect common concerns raised by caregivers of children with autism and frequently addressed by healthcare professionals (e.g., pediatricians, psychiatrists) when parents seek autism-related information: (a) understanding autism (7 questions), (b) diagnosis, risk factors, and causes (15 questions), (c) prognosis and lifespan (4 questions), (d) treatment and interventions (6 questions), (e) skill development and behavior management (4 questions), (f) education and inclusion (3 questions), and (g) daily life and routines (5 questions). These questions were designed to reflect the experiences and information needs of caregivers of children and adolescents with autism, approximately ages 2–18 years. The questions were adapted from prior research evaluating the quality of autism and developmental disability information available on websites and digital platforms and reflect some of the most frequently asked concerns by parents and caregivers (see Appendix A for the complete list [35] and Appendix B for the methodological workflow of the study).

For each AI system, searches were conducted in Turkish only in Türkiye, while English searches were carried out in Türkiye, England, and the USA by four researchers. To minimize personalization effects, new user accounts were created where possible, and browser cache and computer history were cleared before initiating searches. In addition, all searches were conducted in private browsing/incognito mode to reduce the influence of cookies or stored preferences. Questions were entered verbatim, without follow-up prompts, to ensure consistency across tools and conditions. Data collection was completed within a three-day window: Day 1 consisted of ChatGPT queries, Day 2 of Gemini searches, and Day 3 of Microsoft Copilot searches. All responses were downloaded and saved in a spreadsheet for subsequent coding and analysis.

### 2.2. Coding Procedures

Responses generated for each of the 44 questions by ChatGPT, Gemini, and Microsoft Copilot were coded for accuracy, readability, actionability, language use, and reference quality.

**Accuracy**: The evaluation of accuracy followed procedures established in previous studies assessing ChatGPT’s responses to health- and autism-related questions [3,21]. We applied the 3Cs framework, which considers three dimensions: (a) Correctness (the extent to which the information provided is scientifically accurate), (b) Clarity (how clearly the response communicates the information), and (c) Conciseness (the degree to which the response conveys essential knowledge without unnecessary detail or omissions). Each dimension was rated on a four-point scale (4 = completely correct, clear, or concise; 3 = mostly correct, clear, or concise; 2 = somewhat correct, clear, or concise; 1 = completely incorrect, unclear, or not concise). McFayden et al. [3] reported fair to substantial interrater reliability for these dimensions, with Weighted Cohen’s Kappa scores of 0.614 for correctness, 0.455 for clarity, and 0.346 for conciseness. In the present study, accuracy coding was conducted independently by five researchers for each response to the 44 questions across the three AI systems across two languages. All scores were reviewed by the first author to ensure consistency across coders and finalize the dataset. Scores for the three dimensions were averaged to generate an overall accuracy score for each question [3].

**Readability:** Readability of AI-generated responses was assessed using the Flesch–Kincaid Grade Level formula [38], which estimates the U.S. school grade level required to understand a given text based on sentence length and word complexity. Scores on this measure correspond to the following ranges: values between 0 and 3 reflect very basic material suitable for early elementary students (ages 5–8), scores from 3 to 6 correspond to upper elementary levels (ages 8–11), and scores from 6 to 9 indicate middle school readability (ages 11–14). Texts scoring between 9 and 12 fall within the high school range (ages 14–17), scores of 12 to 15 indicate college-level material (ages 17–20), and values above 15 reflect advanced or postgraduate-level texts. For health and caregiver-facing materials, a readability level around grades 6–8 (scores 6–9) is generally recommended [39]. In this study, two researchers independently calculated the Flesch–Kincaid Grade Level for each response, and results were compared to ensure consistency. The Flesch–Kincaid Grade Level is one of the most widely used readability formulas in health communication research and has demonstrated strong validity in predicting text difficulty and reader comprehension [40].

**Actionability:** The actionability of AI-generated responses was evaluated using a single item from the Patient Education Materials Assessment Tool for Printable Materials (PEMAT-P; [41]). The PEMAT-P is a well-established, validated instrument designed to assess how easy educational materials are for lay audiences to understand and how clearly they identify concrete actions that readers can take [3,41]. For the purposes of this study, we applied item #20, which assesses whether the material “clearly identifies at least one action the user can take.” Each response was scored dichotomously (0 = disagree, 1 = agree). By focusing exclusively on this item, our analysis examined the degree to which AI responses offered parents or caregivers concrete, practical next steps related to autism. Actionability coding was carried out for every response across all three AI systems and both languages by two researchers, who independently coded and then reached consensus on the final ratings.

**Language Use:** Language use in AI-generated responses was evaluated to capture both potential bias and alignment with current discussions about terminology within the autism community. Coding drew on recent guidelines that distinguish between medicalized language and neurodiversity-affirming language when describing autism [3]. Each response was rated on a three-point scale: (1) primarily medical language, (2) a mix of medical and neurodiversity-affirming language, or (3) primarily neurodiversity-affirming language. This framework reflects both prior research practices [21] and evolving community perspectives on respectful and accurate ways of discussing autism [3,42]. Previous research has demonstrated that this approach yields reliable results; for example, McFayden et al. [3] reported a weighted Cohen’s Kappa of 0.476 for language coding, indicating moderate agreement among raters and supporting the use of this measure as a dependable coding framework. In the present study, four researchers independently coded language use for each response to the 44 questions generated by the three AI systems in both English and Turkish. Following independent coding, the researchers met to compare ratings and resolve discrepancies. Final ratings were established through consensus.

**References**: For each response, we first recorded the total number of references provided. Each reference was then coded according to its domain extension: .com, .net, .org, .gov, .edu, or other. In addition, every hyperlink was clicked to verify whether it directed the user to an active webpage. This procedure allowed us to evaluate not only the number and type of references generated by the AI systems but also their functional accuracy. When multiple references appeared in a single response, both the count and the distribution of domain types were documented. Results were summarized as the average number of references per response, the proportion of references in each domain category, and the percentage of references that led to active webpages. Prior research has demonstrated that this coding approach is highly reliable; for example, McFayden et al. [3] reported an intraclass correlation coefficient (ICC) of 0.913 for reference coding, supporting the robustness of this measure.

### 2.3. Research Team and Training

The evaluation of responses was carried out by a team of researchers with extensive expertise in autism. The research team included four male and one female researcher, with ages ranging from 32 to 44 years. The first author, who has over 20 years of experience working in the field of autism, led the training process. Drawing on this expertise, the first author provided structured guidance on how to apply the coding frameworks (accuracy, language use, actionability, references, and readability) to ensure consistency and rigor across raters. Among the coding team, three of the four researchers held doctoral degrees in special education with a focus on autism, bringing advanced methodological and clinical knowledge to the evaluation process. The fourth researcher was a doctoral candidate with substantial experience working with families of children with autism and up-to-date expertise in evidence-based practices. Collectively, the team contributed more than 40 years of combined experience working with children with autism and their caregivers, encompassing clinical practice, teacher training, and caregiver support. All researchers were fluent in both English and Turkish, which allowed for consistent and accurate evaluation of responses generated across the two languages. This collective expertise ensured that the coding process was informed by both academic knowledge and practical, real-world experience. The structured training, combined with the diverse yet complementary backgrounds of the researchers, strengthened the reliability and validity of the coding procedures applied in this study.

### 2.4. Interrater Reliability

Responses generated across the 44 questions were coded by multiple researchers depending on the evaluation dimension. For accuracy, all five researchers independently rated each response; for readability, two researchers conducted independent calculations; for actionability, two researchers coded responses; for language, four researchers completed the coding; and for references, two researchers coded responses. Interrater reliability was examined to determine the level of agreement among coders. Intraclass correlation coefficients (ICCs) were calculated for continuous measures (accuracy, readability, and references), while weighted Cohen’s Kappa was used for categorical measures (actionability and language). Any disagreements among coders were resolved through discussion until consensus was reached. Overall, reliability ranged from fair to substantial across coding dimensions. Specifically, ICC values indicated high consistency for accuracy (0.86) and references (0.99), with perfect agreement for readability (1.00). Weighted Cohen’s Kappa values showed substantial agreement for actionability (0.78) and language use (0.74). These coefficients demonstrate that the coding process was highly stable and reproducible across raters and dimensions.

### 2.5. Data Analysis

Data were analyzed using a combination of mixed-design ANOVAs and multilevel modeling (MLM) to evaluate the effects of large language models (LLMs: ChatGPT, Gemini, and Copilot), geographic location (USA, England, Türkiye), and language (English, Turkish) on outcome variables. Accuracy and readability were treated as continuous dependent measures and examined with 2 (Domain Category) × 3 (LLM) × 3 (Location or Language) designs, while actionability, language coding, and references were treated as categorical variables and summarized descriptively. ANOVAs tested main and interaction effects, while MLMs allowed for further examination of the interactions as demonstrated by Field et al. [43]. Effect sizes were reported using generalized eta squared (η^2^) for ANOVAs and *t*-value based effect sizes (r) for significant coefficients (*p* < 0.05) in MLM [43] (p. 641).

To facilitate more interpretable comparisons, the original seven content domains were consolidated into two broader categories. The first, foundational knowledge, included items related to understanding autism as well as its diagnosis, risk factors, and causes (domains a and b; first 22 questions). The second, practical supports, encompassed items addressing prognosis and lifespan, treatment and interventions, skill development and behavior management, education and inclusion, and daily life and routines (domains c through g; last 22 questions). The two-category domain variable constituted the between-subjects factor, whereas LLM and Location constituted the within-subject factor in mixed-design ANOVAs and MLMs. To study the language effect, separate models were conducted by utilizing Language instead of Location as the second within-subjects factor.

## 3. Results

### 3.1. Accuracy

**Figure 1:**
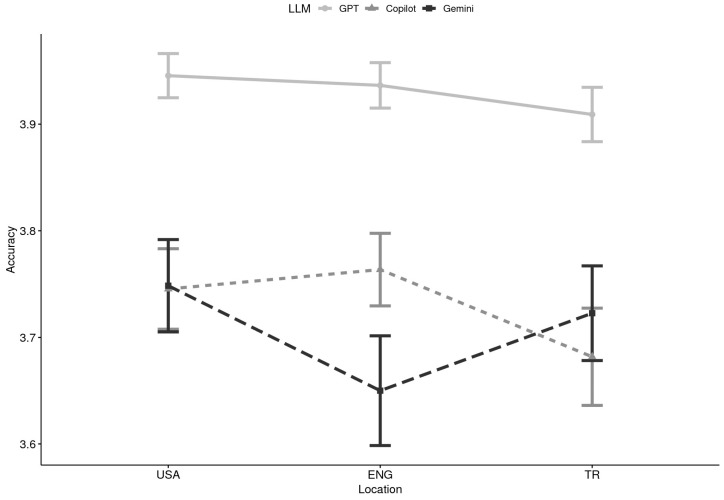
Accuracy by Large Language Model (LLM) and Location (N = 44). Note. USA = United States of America, ENG = England, TR = Türkiye; GPT = ChatGPT.

**Accuracy x Location:** Accuracy scores were consistently high across LLMs, domains, and locations, with means ranging from 3.63 to 3.97 on the 4-point scale (see Table 1). ChatGPT produced the highest accuracy overall, while Copilot yielded the lowest, with Gemini falling in between. For instance, ChatGPT scores were stable across locations (USA: M = 3.97; England: M = 3.94; Türkiye: M = 3.93), whereas Copilot trailed slightly (USA: M = 3.75; England: M = 3.76; Türkiye: M = 3.68). A 2 (Domain category: foundational knowledge vs. practical support) × 3 (LLM: GPT, Gemini, Copilot) × 3 (Location: USA, England, Türkiye) mixed ANOVA revealed a significant main effect of LLM, F(2, 84) = 33.10, *p* < 0.001, *η*^2^ = 0.14, indicating considerable variation in accuracy across the three systems. Domain was not significant, F(1, 42) = 0.10, *p* = 0.748, and Location showed a marginal effect, F(2, 84) = 3.09, *p* = 0.051. Importantly, the LLM × Location interaction was significant, F(4, 168) = 4.12, *p* = 0.003, *η*^2^ = 0.012, while all other interactions were nonsignificant. Follow-up analyses demonstrated that Gemini produced significantly lower accuracy than ChatGPT in England, while Copilot also underperformed relative to ChatGPT in some contexts (see Figure 1).

**Figure 2:**
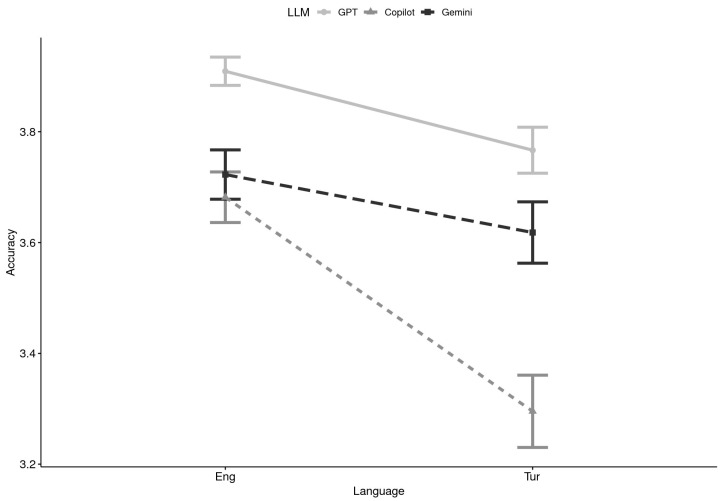
Accuracy by Large Language Model (LLM) and Language (N = 44). Note. Eng = English, Tur = Turkish; GPT = ChatGPT.

**Accuracy x Language:** Within Türkiye, accuracy scores varied as a function of both the LLM and the input language, with overall means again falling in the high range (3.29–3.93 on the 4-point scale; see Table 1). ChatGPT maintained the highest scores in both English and Turkish, while Copilot produced the lowest, especially for Turkish queries (M = 3.30). Gemini performed moderately well but consistently below ChatGPT (Figure 2).

A 2 (Domain category: foundational knowledge vs. practical support) × 3 (LLM: GPT, Gemini, Copilot) × 2 (Language: English vs. Turkish) mixed ANOVA revealed a main effect of LLM, F(2, 84) = 35.77, *p* < 0.001, *η*^2^ = 0.17, indicating clear accuracy differences across systems. A main effect of Language also emerged, F(1, 42) = 50.07, *p* < 0.001, *η*^2^ = 0.10, with English queries producing higher accuracy than Turkish ones. Importantly, the LLM × Language interaction was significant, F(2, 84) = 10.65, *p* = 0.0001, *η*^2^ = 0.04, showing that the accuracy gap between systems differed by language. Domain and all other interactions were nonsignificant.

Multilevel modeling confirmed these findings. Likelihood-ratio tests demonstrated that adding LLM (Δχ^2^ = 53.15, *p* < 0.0001), Language (Δχ^2^ = 43.10, *p* < 0.0001), and their interaction (Δχ^2^ = 19.04, *p* = 0.0001) significantly improved model fit. Fixed-effects estimates indicated that both Copilot (*β* = −0.248, SE = 0.076, *p* = 0.0015, *r* = 0.34) and Gemini (*β* = −0.188, SE = 0.076, *p* = 0.0154, *r* = 0.26) scored significantly lower than ChatGPT overall. Moreover, a Copilot × Turkish interaction (*β* = −0.288, *p* = 0.0036, *r* = 0.26) revealed that compared to ChatGPT, Copilot’s accuracy dropped disproportionately when processing Turkish inputs. The main effect of Turkish compared to English was negative (*β* = −0.103) but did not reach statistical significance (*p* = 0.136).

Taken together, these analyses suggest that English inputs generally elicited more accurate responses than Turkish ones, and that Copilot in particular struggled with Turkish queries, widening the performance gap with GPT. While GPT was consistently accurate across both languages, Gemini and especially Copilot showed reduced performance in Turkish, underscoring the importance of cross-linguistic evaluation in assessing LLM reliability.

### 3.2. Readability (Flesch–Kincaid Grade Level)

**Readability x Location:** Analyses of readability using Flesch–Kincaid Grade Level (FKGL) scores indicated consistent patterns across domains and locations. A 2 (Domain) × 3 (LLM) × 3 (Location) mixed ANOVA revealed a significant main effect of LLM, F(2, 84) = 14.75, *p* < 0.0001, *η*^2^ = 0.053. Neither domain, F(1, 42) = 2.64, *p* = 0.112, nor location, F(2, 84) = 0.12, *p* = 0.888, reached significance, and no interactions were observed. Multilevel modeling produced a convergent pattern of findings. Likelihood ratio tests supported inclusion of LLM (Δχ^2^ = 25.08, *p* < 0.0001), but not domain or location. Fixed-effects estimates indicated that Copilot responses were written at significantly lower grade levels than ChatGPT (*β* = −1.132, *p* = 0.015, *r* = 0.26), suggesting that Copilot produced more accessible content. Gemini responses also trended toward lower readability scores (*β* = −0.850, *p* = 0.065), though this difference did not reach significance.

**Figure 3:**
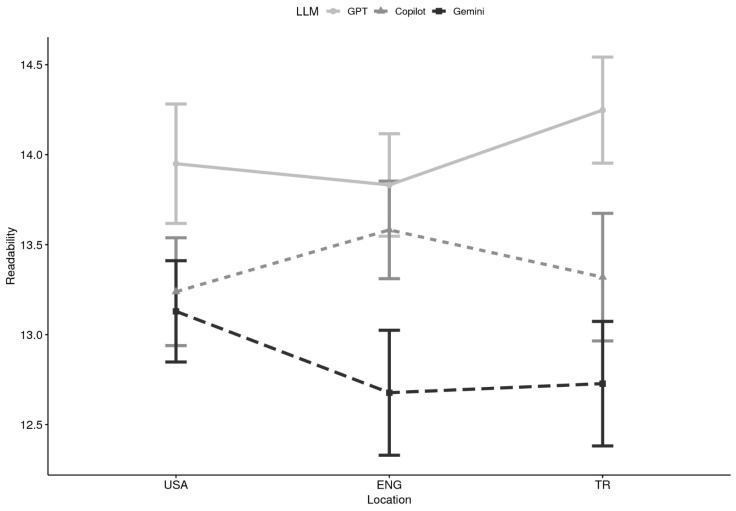
Readability by Large Language Model (LLM) and Location (N = 44). Note. USA = United States of America, ENG = England, TR = Türkiye; GPT = ChatGPT.

Descriptively, ChatGPT responses clustered around grades 13–14 (late high school to early college), whereas Copilot and Gemini produced responses averaging closer to 12–13 (upper secondary; see Table 2 and Figure 3). This pattern suggests that GPT tended to generate more advanced texts, while Copilot and Gemini provided somewhat more accessible responses, though still above the recommended 6th–8th grade level for health materials.

**Figure 4:**
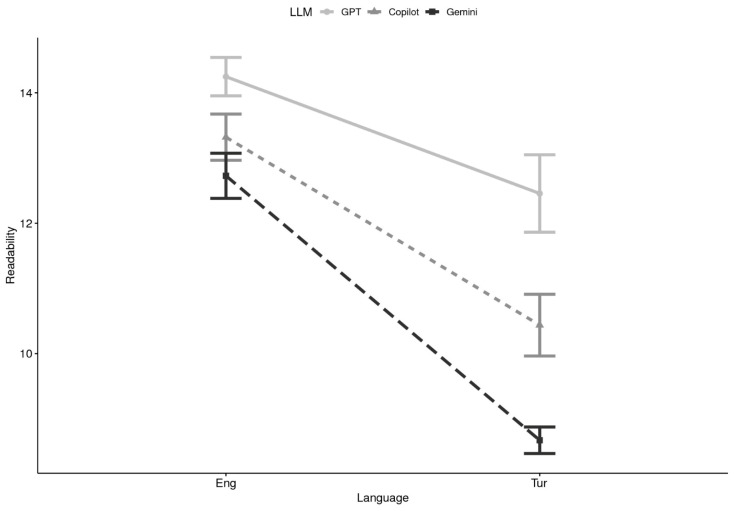
Readability by Large Language Model (LLM) and Language (N = 44). Note. Eng = English, Tur = Turkish; GPT = ChatGPT.

**Readability x Language:** When language was considered, a 2 (Domain) × 3 (LLM) × 2 (Language) ANOVA demonstrated considerable main effects of LLM, F(2, 84) = 26.86, *p* < 0.0001, *η*^2^ = 0.151, and language, F(1, 42) = 53.70, *p* < 0.0001, *η*^2^ = 0.242, as well as a significant LLM × Language interaction, F(2, 84) = 9.41, *p* = 0.0002, *η*^2^ = 0.031. Domain and other interaction terms were nonsignificant. Multilevel models corroborated these results, with significant contributions of LLM (Δχ^2^ = 34.57, *p* < 0.0001), Language (Δχ^2^ = 80.34, *p* < 0.0001), and the LLM × Language interaction (Δχ^2^ = 10.36, *p* = 0.0056). In the fixed-effects solution, Copilot (*β* = −1.470, *p* = 0.043, *r* = 0.22) and Gemini (*β* = −1.958, *p* = 0.008, *r* = 0.29) again produced significantly lower readability scores compared to GPT. The Turkish language main effect (*β* = −1.372, *p* = 0.054) was marginal, indicating a trend toward easier readability in Turkish relative to English. Interaction effects suggested that Gemini, in particular, generated simpler outputs in Turkish than in English (*β* = −1.887, *p* = 0.061). Descriptively, ChatGPT outputs generally fell within the high-school to college range (FKGL 13–14), while Copilot and Gemini produced responses averaging 12–13 in English and somewhat lower in Turkish (see Table 2 and Figure 4). These findings highlight that language strongly influenced readability, with Turkish responses being easier to process across all models, and that Gemini showed the largest drop in grade level when shifting from English to Turkish.

Together, these analyses demonstrate that although all three LLMs produced responses above the recommended 6th–8th grade readability level for health communication, Copilot and Gemini tended to yield more accessible outputs than ChatGPT. Language exerted a particularly strong influence, with Turkish responses rated at lower grade levels than English ones, especially for Gemini. In practical terms, while ChatGPT provided more advanced, academic-style texts, Copilot and Gemini responses were relatively more digestible for lay readers, though still not fully aligned with established health communication guidelines.

### 3.3. Actionability

**Figure 5:**
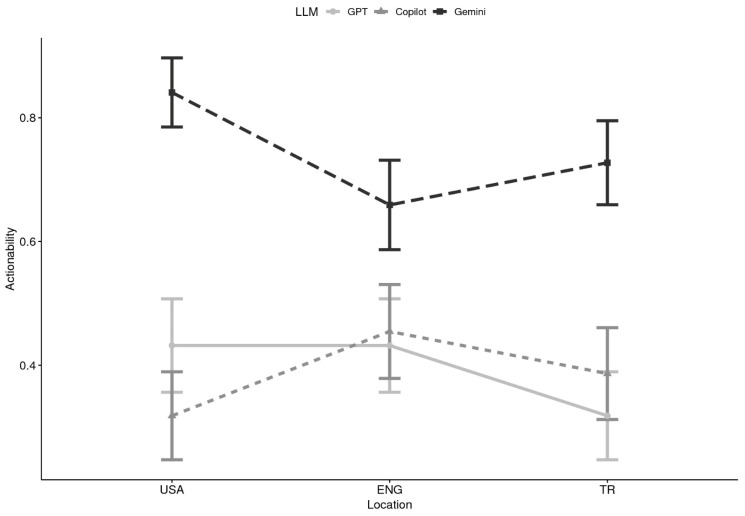
Actionability by Large Language Model (LLM) and Location (N = 44). Note. USA = United States of America, ENG= England, TR = Türkiye; GPT = ChatGPT.

**Actionability x Location:** Descriptive analyses indicated clear differences among LLMs in the likelihood of generating actionable responses. Gemini consistently produced the highest proportion of actionable content across locations. For example, in the USA, Gemini responses were actionable in 84% of cases (M = 0.84), compared to 43% for ChatGPT and 32% for Copilot. In England, Gemini remained higher (M = 0.66) relative to ChatGPT (M = 0.43) and Copilot (M = 0.45). In Türkiye, Gemini again exceeded ChatGPT and Copilot (M = 0.73 vs. M = 0.32 and M = 0.39, respectively). These descriptive patterns suggest a possible LLM × Location interaction, whereby Gemini’s advantage was robust across countries, while GPT and Copilot fluctuated modestly by setting (see Table 3 and Figure 5).

**Figure 6:**
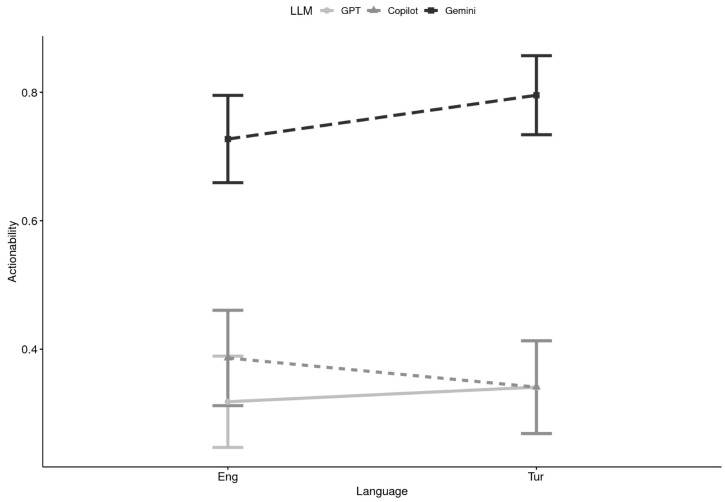
Actionability by Large Language Model (LLM) and Language (N = 44). Note. Note. Eng= English, Tur = Turkish; GPT = ChatGPT.

**Actionability x Language:** When comparing across languages, Gemini again outperformed the other systems (see Table 3 and Figure 6). In English, Gemini responses were actionable 73% of the time (M = 0.73), compared to ChatGPT (M = 0.32) and Copilot (M = 0.39). In Turkish, Gemini was even more likely to provide an explicit user-oriented action (M = 0.80), compared with ChatGPT (M = 0.34) and Copilot (M = 0.34). These findings imply strong main effects of LLM, with Gemini substantially outperforming ChatGPT and Copilot, and only minor variation attributable to language. Domain effects may also be present but were not as visually evident in the descriptive breakdowns.

Across both geographic and linguistic comparisons, Gemini most frequently generated responses that met the PEMAT-P criterion of providing at least one clear, user-oriented action. GPT and Copilot were less likely to do so, with proportions clustering around one-third of responses. These differences appear stable across languages and locations, reinforcing Gemini’s relative strength in guiding users toward actionable next steps.

### 3.4. Language Use

Descriptive analysis of language use revealed distinct patterns across LLMs, locations, and languages (see Table 4). Across all conditions, the majority of AI-generated responses employed primarily medical language, with limited use of neurodiversity-affirming terms.

**Language Use x Location***:* In the USA, ChatGPT and Copilot produced similar distributions, with 61% and 68% of responses, respectively, rated as using primarily medical language (ML), and the remainder showing a mix of medical and neurodiversity-affirming language (NAL). Gemini showed the highest proportion of ML use (71%) in the USA, with only one response (2%) coded as primarily affirming. In England, this pattern persisted. ChatGPT (57% ML), Copilot (71% ML), and Gemini (68% ML) all favored medical terminology, with again only 1–2% of responses showing affirming language. In Türkiye, the trend continued. ChatGPT (64% ML), Copilot (68% ML), and Gemini (66% ML) all generated responses that were predominantly medical in tone. No affirming responses were recorded for any of the LLMs in this condition, though 32–37% of responses showed a blended use of medical and affirming language.

Language Use x Language. Across all three AI systems, Turkish-language responses in the Türkiye condition demonstrated a slightly higher reliance on medical language compared to their English-language counterparts. For example, ChatGPT produced 71% ML responses in Turkish versus 64% in English; Copilot showed a similar increase (77% ML in Turkish vs. 68% in English). Gemini remained consistent across both languages, with 66% ML responses in both. The proportion of neurodiversity-affirming language (NAL) was similar across languages. These results suggest that language of input may influence language style, with Turkish queries resulting in slightly more medicalized framing. Overall, no model consistently prioritized neurodiversity-affirming language across location and languages.

### 3.5. References

**References x Location:** Descriptive analyses revealed substantial variability across LLMs and locations in reference generation (see Table 5). ChatGPT did not produce any references in the USA, England, or Türkiye conditions (M = 0.00). Copilot generated modest numbers of references, with the highest output in England (M = 2.30, SD = 0.70), followed by Türkiye (M = 1.45, SD = 1.07) and the USA (M = 0.75, SD = 1.26). By contrast, Gemini consistently produced the largest volume of references, particularly in the USA (M = 7.36, SD = 4.19) and England (M = 5.82, SD = 4.26), with lower but still notable counts in Türkiye (M = 2.57, SD = 4.44).

Domain-level analyses further highlighted that Gemini produced more references for practical support queries (e.g., USA: M = 9.27, SD = 4.28; England: M = 6.41, SD = 4.18) than for foundational knowledge queries (USA: M = 5.45, SD = 3.16; England: M = 5.23, SD = 4.34). Copilot’s production was more balanced across domains (e.g., England: foundational M = 2.23 vs. practical M = 2.36). Gemini’s reference counts also showed greater variability (SDs up to 5.00), reflecting inconsistent output across items.

When the URL extensions were collapsed into scholarly (.org/.gov/.edu), commercial (.com/.net), and other categories (see Table 6), Gemini consistently produced the largest proportion of scholarly references across locations (USA: 74%, England: 66%, Türkiye: 62%). Copilot leaned more heavily on commercial domains, especially in the USA (58%) and Türkiye (61%), with only England showing a more balanced split (42% scholarly vs. 56% commercial). Gemini also retrieved a broader mix of “other” sources (e.g., .uk, .int, .scot), especially in England (13%), suggesting wider but sometimes less consistent sourcing.

**References × Language:** Patterns by language mirrored those observed by location (see Table 5). ChatGPT again produced no references. Copilot returned relatively few references in either Turkish (M = 0.93, SD = 1.00) or English (M = 1.45, SD = 1.07), while Gemini generated more in both Turkish (M = 1.32, SD = 0.96) and English (M = 2.57, SD = 4.44). For Gemini, domain-level differences were evident: in English queries, more references were produced for foundational knowledge items than practical support ones (M = 3.36 vs. 1.77), while in Turkish, the difference was smaller (M = 1.41 vs. 1.23). Copilot’s output was comparatively balanced across domains in both languages.

URL extension analyses by language further emphasized Gemini’s stronger scholarly referencing (see Table 6). For English queries, Gemini generated 62% scholarly links compared with Copilot’s 36%. For Turkish queries, Gemini again produced more references overall, but the scholarly proportion dropped to 31% compared with Copilot’s 27%, with both systems relying more heavily on commercial sources in this context.

Overall, Gemini was the only LLM to consistently generate a substantial number of references, especially in the USA and England conditions and for English-language queries. Copilot produced some references but at relatively low frequencies, while ChatGPT did not provide any. Gemini also showed stronger alignment with scholarly domains, although its higher variability suggests inconsistency in sourcing. Copilot relied more heavily on commercial domains, particularly outside of England. Across both location and language, Gemini tended to produce more references for practical support queries, but reliability concerns remain given the inconsistent counts across items.

## 4. Discussion

The primary purpose of this study was to evaluate and compare the quality of autism-related information generated by three large language models, ChatGPT, Gemini, and Microsoft Copilot, across different countries (the USA, England, Türkiye) and languages (English and Turkish). By analyzing responses to 44 caregiver-relevant questions across two broad domains (foundational knowledge and practical supports), the study aimed to assess the accuracy, readability, actionability, reference quality, and language framing of AI-generated content. This study was designed to provide a detailed comparative understanding of how leading AI models perform within clearly defined linguistic and geographic boundaries. In doing so, it sought to offer a more comprehensive understanding of the potential role of AI in supporting autism education and family decision-making, particularly as a complement to healthcare providers’ guidance, across linguistic and cultural boundaries.

Findings of this study are especially relevant for healthcare professionals, including pediatricians, child psychiatrists, speech–language pathologists, and developmental specialists, who are often the first point of contact for families concerned about autism [15,16]. Providers are expected not only to deliver diagnostic or therapeutic services but also to act as trusted sources of information, helping parents navigate an increasingly complex landscape of autism-related knowledge [17]. However, limited time during clinical encounters, the high emotional stakes of autism diagnoses, and families’ growing dependence on digital information sources present persistent challenges. Within this context, the present analysis contributes to understanding whether, and under what conditions, AI tools, already used informally by many parents, can be safely integrated into the broader healthcare information ecosystem.

Overall, the findings revealed that ChatGPT consistently produced the most accurate content, with high correctness, clarity, and conciseness scores across all locations and languages. This finding supports the findings of earlier studies [3,21] and strengthens confidence in GPT-based tools as reliable sources of factual health information. However, ChatGPT’s lack of references across all queries, regardless of language or topic, was a notable limitation. In an era where source transparency is crucial for establishing trust, this finding suggests a disconnect between GPT’s informational strength and its verifiability. While users might receive accurate explanations, they are left without means to validate or further explore the information through linked sources. This highlights a key implication for practice: healthcare providers guiding families to use AI tools should encourage them to explicitly request references or source documentation when using ChatGPT. Doing so may enhance the credibility of the information provided, especially when families are using these tools to support decision-making or navigate complex care systems. From a broader perspective, this limitation also relates to the ongoing debate about algorithmic transparency and epistemic trust in AI communication [44,45]. When users cannot see or verify information sources, they must rely on the model’s perceived authority, a dynamic that can reinforce information asymmetry and limit informed decision-making in health contexts.

In contrast, Gemini emerged as the most actionable tool, frequently offering concrete next steps, strategies, or suggestions that users could implement in daily life. This finding is particularly important for caregivers seeking practical guidance on issues like behavior management, daily routines, or service navigation. Healthcare providers could find this feature especially useful when recommending follow-up resources to families outside of office visits. Interestingly, Gemini’s actionability was even stronger in Turkish than in English, a surprising result given that most AI systems are typically optimized for English-language use. This suggests potential strengths in Gemini’s training or search capabilities for certain non-English contexts, though further investigation is warranted. Similar language-related disparities have been identified in other health domains. For instance, Sallam et al. [33] found that generative AI models provided significantly higher quality responses in English compared to Arabic when queried about infectious diseases. Taken together with the present findings, this highlights that language remains a critical determinant of AI output quality, with implications for health equity and access to reliable information in non-English-speaking populations. These differences reflect the phenomenon of linguistic dominance in large language model training, where English-language data are disproportionately represented, leading to structural biases that favor Western-centric discourses and limit global inclusivity [46,47]. As a result, AI tools risk reinforcing existing disparities in knowledge accessibility, particularly for families seeking autism information in underrepresented languages such as Turkish.

Microsoft Copilot presented a more mixed picture. While it generated content with lower readability scores suggesting that its responses were easier to comprehend, it also yielded the lowest accuracy scores across models, particularly for Turkish-language queries. This trade-off reflects a central challenge in AI-mediated health communication: enhancing readability should not come at the expense of factual correctness [26,30]. Notably, Copilot’s performance showed greater variability by language compared to ChatGPT and Gemini, with marked declines in both accuracy and usability for non-English outputs. This pattern aligns with prior research indicating that LLMs generally perform more reliably in English than in other languages such as Arabic or Turkish, due to differences in training data and linguistic optimization [33]. For healthcare professionals and caregivers operating in multilingual contexts, this inconsistency could hinder effective communication and may adversely affect care quality and family decision-making [25]. Beyond technical accuracy, this issue illustrates how AI bias can intersect with linguistic inequity: models trained primarily on Global North datasets may unintentionally encode cultural assumptions, rhetorical styles, and healthcare frameworks that are less applicable or even misleading in non-Western contexts [48]. Addressing such biases requires intentional diversification of training data and evaluation benchmarks that represent the cultural and linguistic diversity of real-world users.

Another unexpected finding involved the use of language framing. Despite growing calls for neurodiversity-affirming communication in autism discourse [42,49], all three models predominantly used medicalized terminology. Very few responses included affirming or strength-based language, and no model consistently prioritized inclusive framing, even in foundational knowledge questions. This pattern persisted across languages and geographic contexts, raising concerns about whether AI tools are keeping pace with evolving community norms around respectful and person-centered language. For example, the AUTALIC dataset reveals that current LLMs often fail to identify ableist or anti-autistic language even when it is subtle or context-dependent [50], and neurodivergent users report that many AI responses reflect assumptions rooted in medicalized norms rather than strengths or identity [51]. For healthcare providers committed to inclusive care, this suggests that LLMs should be used cautiously and ideally supplemented with curated, community-aligned resources. This finding also connects to the broader conversation on cultural inclusivity and representational fairness in AI. When models reproduce dominant medicalized narratives, they perpetuate historical power imbalances in how disability is discussed and understood [52]. Ensuring inclusive AI systems requires both linguistic sensitivity and participatory approaches that involve neurodivergent individuals in the design and evaluation of AI training data and outputs.

In terms of reference generation, Gemini stood out as the only tool that reliably included sources, many of which were from scholarly or reputable domains. However, it also displayed high variability, with some responses offering up to nine references and others providing none. The reliability of reference links and the use of non-standard domains (e.g., .scot, .int, .media) further complicate users’ ability to evaluate source credibility. ChatGPT’s complete omission of references and Copilot’s preference for commercial domains in certain contexts suggest that users should be cautious when relying on these tools for sourcing further information. For healthcare professionals, this finding underscores the need to guide families in verifying AI-generated content, especially when it lacks source transparency. One actionable strategy is to encourage families to prioritize information hosted on websites ending in .gov, .edu, or .org, as these domains are more likely to contain evidence-based and professionally vetted content [36,53]. By reinforcing the value of reputable sources, providers can help mitigate the risk of families acting on incomplete or inaccurate information retrieved from AI-generated outputs [35]. Moreover, the inconsistent referencing patterns observed across tools may reflect deeper structural limitations in how AI systems represent knowledge provenance, raising epistemological questions about what counts as “trusted” information in algorithmic contexts [54]. Future work should therefore focus on designing models that not only generate accurate and culturally sensitive content but also provide transparent, traceable sources that support user trust and critical evaluation.

Taken together, the present findings offer comparative depth on the strengths and weaknesses of leading AI models in generating autism-related information across different languages and settings. While each model has strengths, ChatGPT in accuracy, Gemini in actionability, and Copilot in accessibility, none offer a complete solution. These results highlight the importance of healthcare professionals critically evaluating the capabilities of AI tools before recommending them to families. It is essential to consider that different tools may be more or less appropriate depending on a family’s specific needs, preferred language, and health literacy level. Furthermore, the findings emphasize the need to assess AI-generated content across multiple dimensions, not only for accuracy but also for usability factors that influence how information is interpreted, trusted, and acted upon. Ultimately, this study contributes to ongoing discussions about how AI can either bridge or reinforce existing inequities in global health communication. Ensuring that AI models support, not supplant, cultural diversity, linguistic inclusivity, and neurodiversity-affirming discourse will be critical to advancing equitable and ethical uses of AI in healthcare.

### 4.1. Implications for Practice and Policy

The findings of this study highlight the growing potential of LLMs to complement healthcare professionals’ efforts in autism education and family guidance. As families increasingly turn to AI-driven tools to explore autism-related questions, healthcare providers must be prepared to offer informed recommendations about how to use these systems effectively and safely. Rather than replacing professional advice, LLMs can extend provider communication by offering supplemental information, particularly between visits or in resource-limited settings.

To support families in using these tools responsibly, providers can take several concrete steps. First, when recommending AI tools like ChatGPT, clinicians should encourage families to include explicit prompts requesting references or source links, as ChatGPT typically does not offer these unprompted. Doing so may enhance transparency and foster more critical engagement with the content. Providers should also advise families to prioritize information retrieved from websites ending in .gov, .edu, or .org, as these domains are more likely to contain evidence-based, peer-reviewed, and professionally vetted resources.

Second, providers may consider tailoring their guidance based on each tool’s specific strengths. For example, ChatGPT is especially well-suited for delivering accurate definitions and explanations, making it a helpful option when families are seeking foundational knowledge or clarification of common myths. Gemini, in contrast, is better equipped to generate actionable advice, such as behavior strategies, communication tips, or daily living suggestions, and may be a more useful tool for supporting parents in day-to-day decision-making. Copilot, though less consistent in accuracy, may offer more readable content for some families, particularly those with lower literacy levels or limited English proficiency, but should be used with caution and always in conjunction with verified sources.

Healthcare professionals also play a critical role in setting expectations for AI use. Families should be reminded that LLMs are not substitutes for professional evaluations, individualized interventions, or clinical expertise. Providers can proactively share sample prompts, vetted question templates, or AI literacy resources to help parents formulate more precise and effective queries. These strategies are particularly valuable in underserved communities where access to autism specialists may be limited, and where AI tools could serve as interim support while families await further evaluation or services.

Finally, organizations and practices may benefit from developing quick-reference handouts or digital guides that outline safe and effective AI use for families. These could include tips on evaluating credibility, identifying biased or stigmatizing language, and translating AI responses into discussions with healthcare teams. As AI tools continue to evolve, such resources will be essential to ensure that families are empowered, not misled, by the digital information they encounter.

Beyond clinical application, these findings also have important ethical and policy implications. As AI-generated health information becomes more prevalent, questions of accountability and informed consent become increasingly urgent. When misinformation or biased content is produced by AI systems, it is often unclear who holds responsibility, the developer, the healthcare provider recommending the tool, or the user interpreting it [55,56]. This “delegated trust problem” emphasizes the need for transparent oversight mechanisms that ensure AI models used in healthcare contexts meet established standards of accuracy, privacy, and fairness. Policymakers should consider developing regulatory frameworks similar to medical device approval processes, requiring algorithmic auditing, data provenance disclosure, and ethical labeling for AI health applications. Such measures would help clarify professional liability, safeguard patient autonomy, and promote public trust in the responsible use of AI for health communication and decision support.

At the same time, it is essential to recognize that AI-generated information should be viewed as a supplementary resource rather than a clinical decision-making tool. Overreliance on AI outputs by healthcare providers or families could risk misinformation, misinterpretation, and erosion of medical standards. Therefore, implementation of AI-assisted content in healthcare should occur only under human supervision and within established professional and ethical boundaries [55,56]. Clear institutional policies and clinician training on appropriate AI use can help ensure that these technologies enhance, rather than replace, clinical judgment and patient-centered care.

Importantly, although this study examined only three countries and two languages, this multi-country, bilingual design offers broader generalizability than most prior investigations, which have been confined to single-language or single-context analyses [3,33]. By assessing English-language outputs across three distinct national contexts and comparing them with Turkish-language data, this study provides a more comprehensive picture of how AI-generated autism information performs across diverse healthcare and cultural systems. These findings thus extend the scope of current research beyond national boundaries, highlighting both shared strengths and language-specific gaps in AI-mediated health communication.

### 4.2. Limitations and Recommendation for Future Research

While this study provides valuable insights into the use of AI tools for autism-related information, several limitations should be noted. First, only three LLMs (ChatGPT, Gemini, and Copilot) were examined. Future research should extend this work by including other models such as DeepSeek and Llama which are increasingly shaping the AI landscape and may offer distinct linguistic or contextual advantages across healthcare domains. Second, all queries were entered as single-turn prompts. Although this approach ensured methodological consistency and allowed for direct, standardized comparison across tools, languages, and countries, it does not fully capture how users typically engage in multi-turn, interactive conversations. Multi-turn exchanges can influence the quality and personalization of AI responses, but including them in this study would have introduced user-driven variability that could not be systematically controlled. Therefore, the single-turn design was intentionally chosen to evaluate the baseline informational quality of each model under comparable and replicable conditions. Future studies should build on this work by examining how LLMs perform in dynamic, real-world dialogue scenarios where conversational context and iterative questioning may further shape response quality and accuracy. Third, the study included only English and Turkish responses. While this allowed for meaningful cross-linguistic comparisons, future research should examine additional languages such as Spanish, Arabic, and Mandarin to explore generalizability and cultural relevance in diverse settings. Finally, this study assessed content quality but not behavioral outcomes. Future research should explore whether LLM-generated information actually influences family decisions, service use, or child outcomes.

While this study focused on three countries (the United States, England, and Türkiye) and two languages (English and Turkish), this design represents a substantial methodological advancement over most prior studies, which have typically examined AI-generated health information within a single linguistic or geographic context [3,33]. By evaluating English-language outputs across multiple national settings and including a non-English language comparison, this study captures both linguistic and regional variability, offering a stronger basis for generalization to healthcare contexts than prior single-nation studies. Future research may expand this cross-context approach to include additional languages and regions to further strengthen external validity and global applicability.

## 5. Conclusions

This study provides a detailed comparative evaluation of how AI-driven language models perform in generating autism-related information across different languages, countries, and content domains. The findings showed that ChatGPT consistently produced the most accurate responses across all locations and languages, followed by Gemini and Copilot, with only minor variability by setting. These results reinforce the potential of GPT-based systems as factually reliable sources of educational content for families and healthcare professionals [RQ1: Accuracy × Location/Language]. Across all models, the readability of responses remained above the recommended sixth- to eighth-grade level for health materials. While Copilot generated the most accessible text and Gemini followed closely, ChatGPT produced more complex responses, highlighting that readability continues to pose a challenge in AI-generated health communication [RQ2: Readability differences across tools/languages].

Gemini, however, provided the most actionable and user-oriented responses, frequently suggesting practical steps or strategies that caregivers could apply—an advantage particularly evident in Turkish-language outputs [RQ3: Actionability]. In terms of language framing, all three models relied heavily on medicalized terminology, with limited use of neurodiversity-affirming or strengths-based language. This indicates a persistent gap between AI-generated content and current best practices in inclusive communication [RQ4: Language framing (medicalized vs. neurodiversity-affirming)]. When considering reference generation, Gemini again stood out for consistently including credible sources, while Copilot showed mixed performance and ChatGPT omitted references unless explicitly requested [RQ5: References; frequency, credibility, functionality].

Together, these findings demonstrate that while AI tools have clear strengths in accuracy, accessibility, and practical guidance, they also exhibit notable weaknesses in inclusivity, readability, and source transparency. These results highlight the importance of healthcare professionals guiding families in how to use AI responsibly and critically. As AI technologies continue to evolve, their integration into health communication must be accompanied by ongoing evaluation, professional oversight, and ethical safeguards to ensure that digital tools complement, rather than replace, human-centered care for autistic individuals and their families.

## Figures and Tables

**Table 1:** Descriptive Statistics for Accuracy by Large Language Model, Location, Domain Category and Language (N = 44).

LLM	Location/Language	Category	Median	Mean	SD
ChatGPT	USA/English	Foundational	4.00	3.97	0.06
Copilot	USA/English	Foundational	3.80	3.75	0.25
Gemini	USA/English	Foundational	3.87	3.78	0.27
ChatGPT	England/English	Foundational	4.00	3.94	0.09
Copilot	England/English	Foundational	3.83	3.76	0.22
Gemini	England/English	Foundational	3.73	3.63	0.35
ChatGPT	Türkiye/English	Foundational	4.00	3.93	0.11
Copilot	Türkiye/English	Foundational	3.73	3.68	0.28
Gemini	Türkiye/English	Foundational	3.83	3.75	0.29
ChatGPT	USA/English	Practical	4.00	3.92	0.18
Copilot	USA/English	Practical	3.80	3.74	0.26
Gemini	USA/English	Practical	3.87	3.72	0.31
ChatGPT	England/English	Practical	4.00	3.94	0.18
Copilot	England/English	Practical	3.87	3.76	0.24
Gemini	England/English	Practical	3.77	3.67	0.35
ChatGPT	Türkiye/English	Practical	4.00	3.88	0.21
Copilot	Türkiye/English	Practical	3.77	3.68	0.33
Gemini	Türkiye/English	Practical	3.80	3.70	0.30
ChatGPT	Türkiye/Turkish	Foundational	3.87	3.83	0.17
Copilot	Türkiye/Turkish	Foundational	3.33	3.29	0.41
Gemini	Türkiye/Turkish	Foundational	3.70	3.58	0.37
ChatGPT	Türkiye/Turkish	Practical	3.77	3.70	0.34
Copilot	Türkiye/Turkish	Practical	3.30	3.30	0.47
Gemini	Türkiye/Turkish	Practical	3.83	3.65	0.37

**Table 2:** Descriptive Statistics for Readability by Large Language Model, Location, Domain Category and Language (N = 44).

Location/Language	Category	Median	Mean	SD
USA/English	Foundational	13.70	13.82	1.61
USA/English	Foundational	12.50	12.69	1.84
USA/English	Foundational	13.25	12.97	1.92
England/English	Foundational	13.65	13.84	1.29
England/English	Foundational	13.05	13.21	1.73
England/English	Foundational	11.45	11.89	2.59
Türkiye/English	Foundational	14.40	14.13	1.68
Türkiye/English	Foundational	12.10	12.66	2.23
Türkiye/English	Foundational	11.95	12.17	1.78
USA/English	Practical	14.55	14.08	2.70
USA/English	Practical	14.15	13.79	2.02
USA/English	Practical	13.55	13.29	1.84
England/English	Practical	13.30	13.83	2.37
England/English	Practical	14.15	13.96	1.83
England/English	Practical	13.60	13.46	1.69
Türkiye/English	Practical	14.70	14.37	2.23
Türkiye/English	Practical	13.75	13.98	2.33
Türkiye/English	Practical	13.05	13.29	2.64
Türkiye/Turkish	Foundational	12.77	12.76	4.27
Türkiye/Turkish	Foundational	9.50	10.06	2.04
Türkiye/Turkish	Foundational	8.84	8.91	1.25
Türkiye/Turkish	Practical	10.43	12.16	3.64
Türkiye/Turkish	Practical	10.12	10.82	3.97
Türkiye/Turkish	Practical	8.31	8.43	1.42

**Table 3:** Descriptive Statistics for Actionability by Large Language Model, Location, and Language (N = 44).

LLM	Location/Language	Median	Mean	SD
ChatGPT	USA/English	0.00	0.43	0.50
Copilot	USA/English	0.00	0.32	0.47
Gemini	USA/English	1.00	0.84	0.37
ChatGPT	England/English	0.00	0.43	0.50
Copilot	England/English	0.00	0.45	0.50
Gemini	England/English	1.00	0.66	0.48
ChatGPT	Türkiye/English	0.00	0.32	0.47
Copilot	Türkiye/English	0.00	0.39	0.49
Gemini	Türkiye/English	1.00	0.73	0.45
ChatGPT	Türkiye/Turkish	0.00	0.34	0.48
Copilot	Türkiye/Turkish	0.00	0.34	0.48
Gemini	Türkiye/Turkish	1.00	0.80	0.41

**Table 4:** Descriptive Statistics for Language Use by Large Language Model, Location, and Language (N = 44).

LLM	Location/Language	ML	Mix of ML/NAL	NAL
ChatGPT	USA/English	27 (61%)	17 (39%)	0
Copilot	USA/English	30 (68%)	14 (32%)	0
Gemini	USA/English	31 (71%)	12 (27%)	1 (2%)
ChatGPT	England/English	25 (57%)	18 (41%)	1 (2%)
Copilot	England/English	31 (71%)	13 (29)	0
Gemini	England/English	30 (68%)	13 (29)	1 (2%)
ChatGPT	Türkiye/English	28 (64%)	16 (37%)	0
Copilot	Türkiye/English	30 (68%)	14 (32%)	0
Gemini	Türkiye/English	29 (66%)	15 (34%)	0
ChatGPT	Türkiye/Turkish	31 (71%)	12 (27%)	1 (2%)
Copilot	Türkiye/Turkish	34 (77%)	9 (21%)	1 (2%)
Gemini	Türkiye/Turkish	29 (66%)	13 (29)	2 (5%)

Note. ML = Medical Language; NAL = Neurodiversity-Affirming Language.

**Table 5:** Descriptive Statistics for References by Large Language Model, Location, Language, and Domain.

LLM	Location/Language	Category	Frequency	Mean	SD
ChatGPT	USA/English	Foundational	0	0	0
Copilot	USA/English	Foundational	26	1.18	1.47
Gemini	USA/English	Foundational	120	5.45	3.16
ChatGPT	England/English	Foundational	0	0	0
Copilot	England/English	Foundational	49	2.23	0.61
Gemini	England/English	Foundational	115	5.23	4.34
ChatGPT	Türkiye/English	Foundational	0	0	0
Copilot	Türkiye/English	Foundational	34	1.55	1.10
Gemini	Türkiye/English	Foundational	74	3.36	5.00
ChatGPT	USA/English	Practical	0	0	0
Copilot	USA/English	Practical	7	0.32	0.84
Gemini	USA/English	Practical	204	9.27	4.28
ChatGPT	England/English	Practical	0	0	0
Copilot	England/English	Practical	52	2.36	0.79
Gemini	England/English	Practical	141	6.41	4.18
ChatGPT	Türkiye/English	Practical	0	0	0
Copilot	Türkiye/English	Practical	30	1.36	1.05
Gemini	Türkiye/English	Practical	39	1.77	3.74
ChatGPT	Türkiye/Turkish	Foundational	0	0	0
Copilot	Türkiye/Turkish	Foundational	23	1.05	1.05
Gemini	Türkiye/Turkish	Foundational	31	1.41	0.91
ChatGPT	Türkiye/Turkish	Practical	0	0	0
Copilot	Türkiye/Turkish	Practical	18	0.82	0.96
Gemini	Türkiye/Turkish	Practical	27	1.23	1.02

**Table 6:** Descriptive Statistics for URL Extension by Large Language Model, Location, and Language.

**LLM**	**Location/** **Language**	**Total # of References**	**Scholarly**	**Commercial**	**Other**	**Foundational Mean (SD)**	**Practical** **Mean (SD)**
Copilot	USA/English	33	19	14	0	1.18 (1.47)	0.32 (0.94)
Gemine	USA/English	324	79	239	6	5.45 (3.16)	9.27 (4.28)
Copilot	England/English	101	57	42	2	2.23 (0.61)	2.36 (0.79)
Gemine	England/English	256	54	168	34	5.23 (4.34)	6.41 (4.18)
Copilot	Türkiye/English	64	39	23	2	1.55 (1.10)	1.36 (1.05)
Gemine	Türkiye/English	113	39	70	4	3.36 (5.00)	1.77 (3.74)
Copilot	Türkiye/Turkish	41	30	11	0	1.05 (1.05)	0.82 (0.96)
Gemine	Türkiye/Turkish	58	38	18	2	1.41 (0.91)	1.23 (1.02)

Note. ChatGPT did not generate any references across countries or languages and was therefore excluded from this table. Scholarly references include domains ending in .org, .gov, or .edu; commercial references include domains ending in .com or .net; and other references include domains such as .int, .scot, .ie, .in, .me, and .media. # = number.

**Figure A1:**
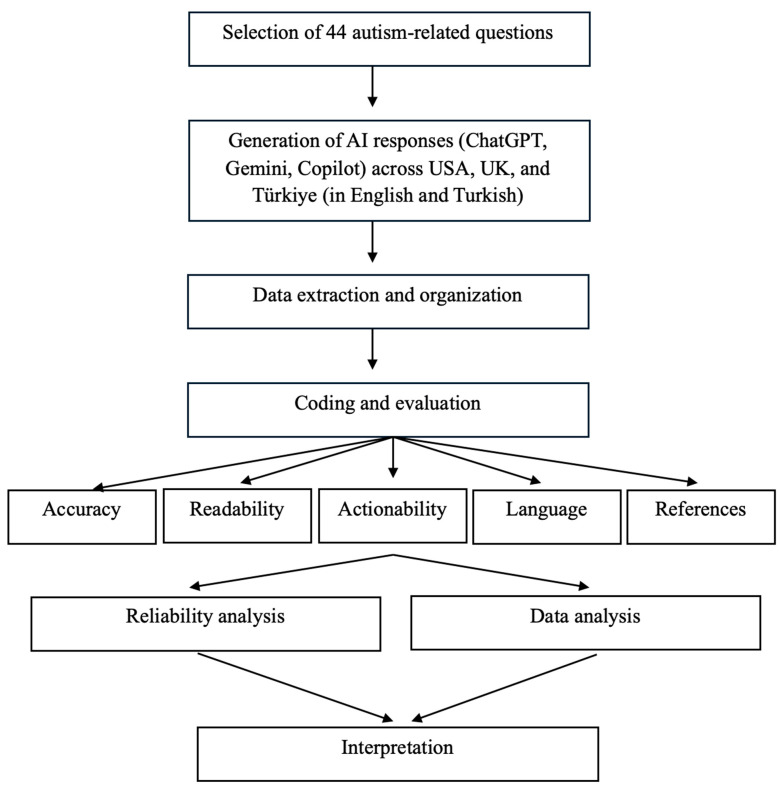
Methodological Workflow of the Study.

## Data Availability

The raw data supporting the conclusions of this article will be made available by the authors on request.

## Appendix A

**Foundational Knowledge**

A. Understanding Autism
  1. What is autism?
  2. When does autism appear?
  3. When is autism diagnosed at the earliest?
  4. What are the early signs of autism?
  5. What are the characteristics of children with autism?
  6. What are the different levels of autism?
  7. What is the prevalence of autism?
B. Diagnosis, Risk Factors, and Causes
  8. How is autism diagnosed?
  9. Can autism be diagnosed during pregnancy?
  10. Do children with autism also have intellectual disability?
  11. Can children with autism speak?
  12. What are the causes of autism?
  13. Is there a specific gene that causes autism?
  14. Are environmental factors such as exposure to pollutants linked to autism?
  15. Does parental age increase the risk of having a child with autism?
  16. Do vaccines cause autism?
  17. Does stress during pregnancy cause autism?
  18. Does medication use during pregnancy cause autism?
  19. Does premature birth increase the risk of autism?
  20. Does watching TV/tablet/phone cause autism?
  21. If there is a child with autism in the family, is my newborn at risk for autism?
  22. Are twins more likely to both have autism?

**Practical Support**

C. Prognosis and Lifespan
  23. Is autism a lifelong condition?
  24. Is there a cure for autism?
  25. Can children with autism outgrow the condition?
  26. Can children with autism become independent?
D. Treatment and Interventions
  27. What treatments are available for children with autism?
  28. What are evidence-based or scientifically supported practices in autism?
  29. What is applied behavior analysis? Is it effective for children with autism?
  30. What role does medication play in managing autism symptoms?
  31. Do special diets (like gluten- and casein-free diets) reduce autism symptoms?
  32. Are therapy animals like dogs, horses, or dolphins effective in reducing autism symptoms?
E. Skill Development and Behavior Management
  33. What can be done to support the social skills of children with autism?
  34. How can problem behaviors be reduced in children with autism?
  35. How can tantrums in children with autism be managed?
  36. How can children with autism be taught to express their emotions?
F. Education and Inclusion
  37. Can children with autism attend general education schools?
  38. What are the challenges of integrating children with autism into general education classrooms?
  39. What benefits does special education offer for children with autism?
G. Daily Life and Routines
  40. What can be done at home to support children with autism?
  41. How should the daily routine of children with autism be organized?
  42. How can the sleep patterns of children with autism be improved?
  43. What should be considered in the diet of children with autism?
  44. What are effective strategies for managing transitions for children with autism?
